# Tea consumption is associated with a reduced risk of high-altitude pulmonary hypertension among high-altitude permanent inhabitants in the Tibetan population: a case-control study

**DOI:** 10.3389/fnut.2026.1732242

**Published:** 2026-02-10

**Authors:** Wuyang Tong, Ran Cheng, Xiaoming Chen, Xuesen Zhang, Wanmin Li, Xiaqing Luo, Shipeng Xu, Xianjin Bi

**Affiliations:** Department of Cardiovasology, People's Liberation Army (PLA) Joint Logistic Support Force 945th Hospital, Ya'an, China

**Keywords:** high-altitude illness, high-altitude pulmonary hypertension, pulmonary hypertension, tea consumption, Tibetan tea

## Abstract

**Background:**

This study investigated the potential association between tea consumption and high-altitude pulmonary hypertension (HAPH) risk in a long-term, high-altitude Tibetan population, which remained unexplored.

**Methods:**

In a hospital-based case-control study, 113 patients with HAPH and 113 controls were included. Data were collected from medical records and a tea consumption questionnaire. Group comparisons were performed using *t*-tests, Mann–Whitney U, Chi-square, or Fisher's exact test. Univariate and multivariable logistic regression analyses determined the tea–HAPH relationship (*p* < 0.05).

**Results:**

Patients with HAPH exhibited significant right-heart structural alterations and a distinct metabolic profile characterized by lower lipid and glucose levels. A significant inverse association was observed between tea consumption and HAPH risk. Compared to the control group, patients with HAPH exhibited a significantly lower proportion of tea consumption (45.1% vs. 59.3%, *p* = 0.033). After adjusting for confounders, including age, hemodynamic, and metabolic parameters, regular tea consumption remained an independent protective factor (adjusted OR = 0.496, 95% CI: 0.258–0.952). Tibetan tea exhibited the strongest protective effect (adjusted OR = 0.300, 95% CI: 0.123–0.735). A significant dose-response relationship was observed, with the significant risk reduction at higher consumption frequency (≥6 days/week: adjusted OR = 0.208), more tea consumption (≥3 cups/day: adjusted OR = 0.305), and longer duration (≥20 years: adjusted OR = 0.210).

**Conclusion:**

Regular Tibetan tea consumption significantly reduces HAPH risk in a dose-response manner. These findings offer new insights into dietary factors in HAPH etiology and can explain Tibetan adaptation to high altitudes.

## 1 Introduction

Over 81.6 million people reside at altitudes above 2,500 meters (1.07% of the total population), primarily in mountainous geographic locations, including Tibet, the Andes, the Ethiopian Highlands, the Pamir, and the Tian-Shan. China has the largest absolute population of approximately 3.5 million (1). Compared with plain areas, the permanent residents of the plateau are at risk of severe health issues due to the hypoxic, low-temperature, and low-pressure environment of high-altitude regions. Chronic hypobaric hypoxia induces vasoconstriction, resulting in pulmonary vascular remodeling and the development of pulmonary hypertension (PH) (2). High-altitude pulmonary hypertension (HAPH) caused by chronic exposure to high altitude is the third clinical classification of PH (3). Our understanding of HAPH remains limited. A study conducted on the Kyrgyz population demonstrated that the incidence of HAPH can reach as high as 14% among long-term residents of high-altitude regions who cannot adapt to chronic hypoxia (4). In South America, the prevalence of HAPH ranges from 5% to 18% (5). Additionally, the prevalence of HAPH among individuals residing at an altitude of 3,250 meters in the Central Asian Plateau is approximately 35% (6). However, HAPH is relatively rare among Tibetan people (7).

Tea, the oldest and most popular beverage worldwide, has deep roots in China, which is both a major producer and consumer. The tea-drinking practices of Tibetan residents are particularly prominent, with a history spanning over 1,000 years and a deep-seated cultural affinity for tea. Tea contains abundant nutrients, including tea polyphenols, organic acids, enzymes, aromatic substances, proteins, pectin, sugars, alkaloids, and amino acids (8). Previous studies have demonstrated that tea contains bioactive components with various health benefits, including cardiovascular protection, antioxidant, anti-inflammatory, anti-sugar, anti-cancer, anti-bacterial, and anti-obesity properties (9–15). Biomedical advances over the last decade have identified the central role of proliferative pulmonary arterial smooth muscle cells (PASMCs) in the development of PH. Epigallocatechin-3-gallate, an efficient antiproliferative compound in tea, has been demonstrated to inhibit PASMCs proliferation in rat models of PH (16–18).

To date, the health benefits of tea consumption have been extensively studied; however, no research has specifically investigated the potential protective relationship between tea consumption and HAPH. We hypothesize that tea consumption can similarly reduce the risk of HAPH in the human population. The relatively low incidence of HAPH in the Tibetan population can be attributed to multiple factors associated with tea consumption, including the type of tea or the amount consumed. In the present study, we included HAPH and non-Patients with HAPH to investigate the association between tea consumption and HAPH risk in the study population.

## 2 Materials and methods

### 2.1 Participants and public involvement statement

The sample size was calculated *a priori* using PASS software (version 15.0, Utah, USA) for a case-control design. With an alpha of 0.05 (two-sided), power of 0.90, a control exposure rate of 60%, 16 and an expected OR of 0.4, a total of 204 participants (102 cases and 102 controls) were required for a 1:1 design. This study enrolled Tibetan patients who were hospitalized at People's Liberation Army (PLA) Joint Logistic Support Force 945th Hospital and The General Hospital of Western Theater Command between January 2020 and December 2024. All participants were required to have been born and permanently reside in high-altitude areas (altitude ≥3,500 m, with a residence duration of ≥20 years). The altitude of each participant's primary residence was ascertained through a two-step verification process. Initially, the participant's place of birth, residential address, and postal code were retrieved from the hospital management system. These data were cross-referenced with the altitude query functions of Baidu Maps and the Geographic Science Data Platform of the Chinese Academy of Sciences to preliminarily identify individuals who were born and resided at an altitude of ≥3,500 meters. Subsequently, confirmation was obtained through outpatient or telephone follow-ups, ensuring that the participants had maintained a stable, permanent long-term residence (≥20 years) in the aforementioned high-altitude areas without migration to lower altitudes. A visual representation of the study area is provided in Supplementary Figure 1 (Geographic distribution and altitude of participant recruitment sites). This visualization was created using Datawrapper (https://www.datawrapper.de/).

Based on the Cologne Consensus Conference 2011, which updated the European Society of Cardiology and European Respiratory Society Guidelines on the Diagnosis and Treatment of PH, and Chemla's evaluation of various empirical formulas for estimating mean pulmonary artery pressure using systolic pulmonary artery pressure (sPAP), we defined PH as an sPAP ≥ 36 mmHg, as estimated by transthoracic echocardiography (TTE). TTE is a well-established non-invasive method for estimating sPAP (19, 20). Multiple studies have demonstrated that sPAP estimated by TTE is highly consistent with measurements obtained through right heart catheterization (21, 22).

**Figure 1:**
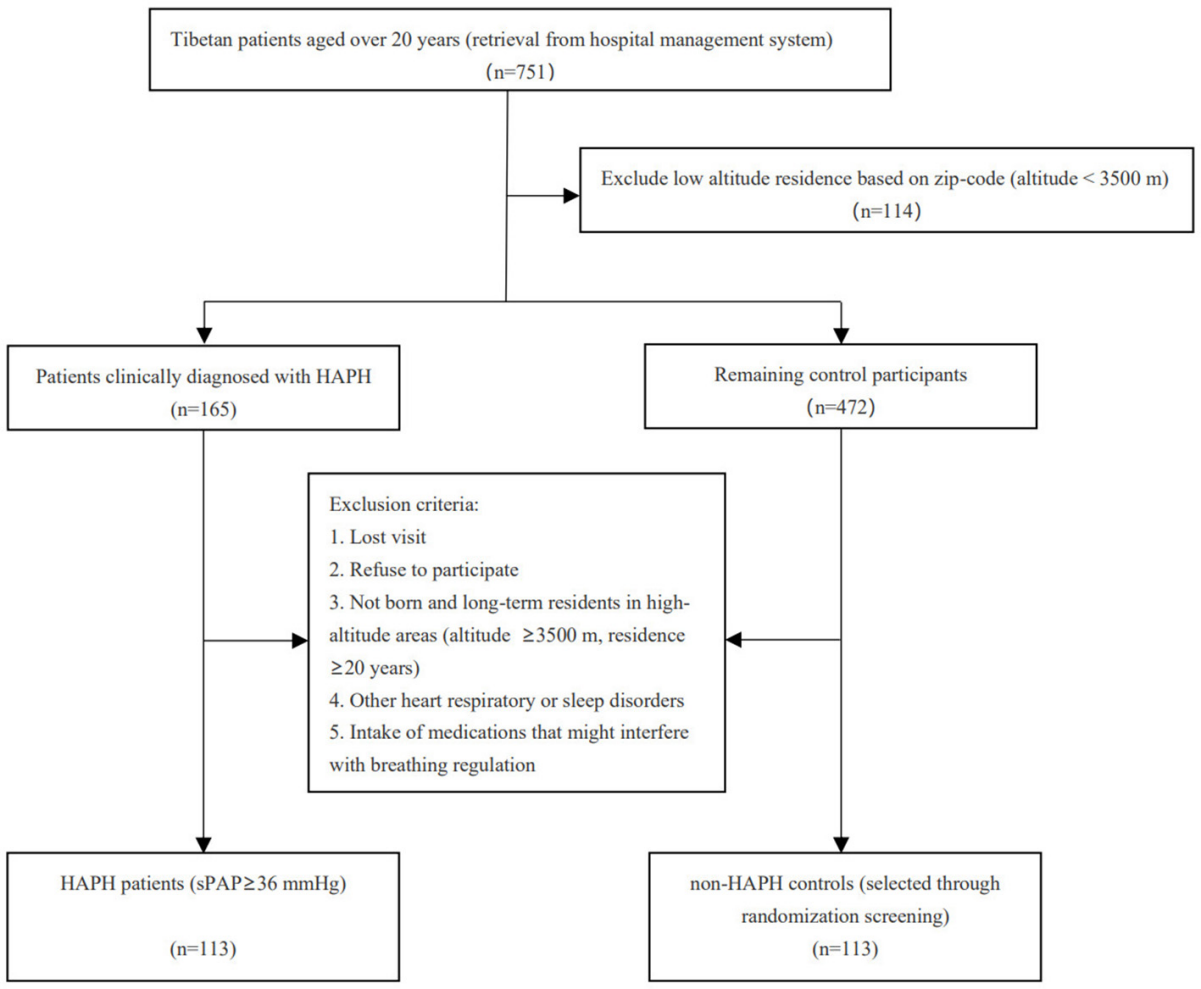
Study flow chart. HAPH, High altitude pulmonary hypertension.

The exclusion criteria included forms of PH other than HAPH, the presence of additional cardiorespiratory or sleep disorders, and the regular intake of medications that could interfere with breathing regulation. A total of 113 patients with clinically diagnosed HAPH were enrolled as the case group. From an initial pool of 472 potential control participants, 126 individuals were excluded based on predefined exclusion criteria, resulting in 346 eligible non-HAPH participants for further selection. To enhance comparability between groups, we applied a matched randomization approach to select 113 non-HAPH controls, individually matched to each HAPH patient at a 1:1 ratio. Matching was primarily based on residence altitude, as the most relevant environmental exposure factor associated with HAPH. For each HAPH case, one control was randomly selected from a pool of candidates with the closest residential altitude (i.e., within a ± 500-meter range or the smallest altitude difference). This process yielded a final control group consisting of 113 participants, ensuring balanced exposure to high-altitude living conditions between the two groups (Figure 1).

All participants provided written informed consent for participation and the subsequent use of their data for analysis. The study adhered to the guidelines of the Declaration of Helsinki. It was approved by the ethics committee of the 945th Hospital of the PLA Joint Logistic Support Force (Approval reference: No. YJGLKT2025K026R003).

### 2.2 Assessment of baseline characteristics

Data were collected from the medical records of patient and control groups, including the following: (I) demographics and baseline clinical characteristics [gender, age, residence, body mass index (BMI), blood pressure, smoking status, alcohol use, and histories of hypertension and diabetes]; (II) laboratory test results (complete blood count, liver and kidney function parameters, and serum metabolic indicators); (III) TTE results (various echocardiographic indices).

### 2.3 Tea consumption assessment

To determine tea consumption patterns, we specifically developed a detailed structured questionnaire for the high-altitude Tibetan population. The questionnaire consists of structured closed-ended questions with standardized response options and was reviewed by a panel of clinical and nutritional experts. It was administered uniformly by trained staff to ensure consistency in data collection. The main content of the questionnaire is as follows: 1. Do you often drink tea? (Yes/No). 2. What types of tea do you drink? (Select from the following: Green tea, Black tea, and Tibetan tea). 3. How many days do you drink tea on average per week? (Select from the following: 1–3 days, 4–5 days, and ≥ 6 days). 4. How many cups of tea do you drink per day? (Select from the following: 1–2 cups, ≥3 cups) 5. How many years have you been drinking tea in the past? (Select from the following: 0–10 years, 10–20 years, and ≥ 20 years). One cup of tea was defined as approximately 200 ml of brewed tea, as clarified to participants during the structured interview. The test-retest reliability of this questionnaire was validated in a separate study (*n* = 60). Administered twice with a 7-day interval, it showed substantial agreement for regular consumption (κ = 0.82) and tea type (κ = 0.78), and good reliability for frequency, amount, and duration (weighted κ = 0.71–0.80).

### 2.4 Statistical analysis

The normality of continuous variables was determined using the Kolmogorov–Smirnov test. Continuous variables are expressed as mean ± standard deviation and analyzed using Student's independent *t*-test or Mann–Whitney U test (if normality assumptions were not met). Categorical variables are expressed as numbers and percentages and analyzed using the chi-squared test or Fisher's exact test (if expected values were ≤ 5). The association between HAPH and tea consumption was determined through univariate and multivariate logistic regression analyses. Initially, we performed univariate logistic regression analysis to identify potential risk factors for HAPH. Variables with a statistical significance level of *p* ≤ 0.2 identified in the univariate logistic regression analysis were further evaluated in the multivariate logistic regression analysis using a backward stepwise method. To evaluate the *p*-values, tea consumption was individually incorporated into logistic regressions, adjusting for variables exhibiting a statistical significance of ≤ 0.2 in the univariate logistic regression analysis. Before constructing the multivariable logistic regression model, we determined multicollinearity among all included independent variables using the variance inflation factor (VIF). A VIF value exceeding 10 is generally regarded as indicative of severe multicollinearity. In this study, all VIF values were well below this conservative threshold, suggesting no significant multicollinearity issues. Statistical analysis was performed using the Statistical Package for Social Sciences (version 28, IBM Corp., Armonk, NY, USA), with significance set at *p* ≤ 0.05.

## 3 Results

### 3.1 Baseline characteristics of the study population

A total of 226 individuals, including 113 patients with HAPH and 113 non-HAPH individuals, were enrolled in this study (Figure 1). The baseline characteristics of the two study populations are summarized in Table 1. Overall, the baseline characteristics of the two groups were largely comparable. However, the mean age of the HAPH group (63.29 ± 14.45 years) was higher than that of the non-HAPH group (55.00 ± 14.31 years). Additionally, the HAPH group exhibited a higher diastolic blood pressure than the non-HAPH group.

**Table 1:** Baseline characteristics of the two study populations.

**Characteristics**	**HAPH**				**non-HAPH**	***P*-value**
	**(*****n*** = **113)**				**(*****n*** = **113)**	
	**Total**	**sPAP** ≥**60 mmHg**	**60 mmHg** > **sPAP** ≥**36 mmHg**	***P*** **-value**	**sPAP** > **36 mmHg**	
	**(*****n*** = **113)**	**(*****n*** = **33)**	**(*****n*** = **80)**		**(*****n***=**113)**	
Age, years	63.29 ± 14.45	63.52 ± 12.97	63.20 ± 15.09	0.857	55.00 ± 14.31	< 0.001^*^
Gender, *n* (%)				0.505		0.789
Male	50 (44.2%)	13 (39.4%)	37 (46.3%)		52 (46.0%)	
Female	63 (55.8%)	20 (60.6%)	43 (53.7%)		61 (54.0%)	
BMI, kg/m^2^	24.71 ± 4.73	23.62 ± 3.83	25.16 ± 5.01	0.225	25.49 ± 4.81	0.145
SBP, mmHg	129.51 ± 17.92	124.52 ± 19.10	131.58 ± 17.11	0.057	126.14 ± 19.91	0.182
DBP, mmHg	84.46 ± 13.88	81.09 ± 14.38	85.85 ± 13.52	0.098	80.51 ± 13.57	0.032^*^
MAP, mmHg	99.48 ± 13.84	95.57 ± 14.56	101.09 ± 13.29	0.053	95.72 ± 14.73	0.050
History of smoking, *n* (%)	22 (19.5%)	10 (30.3%)	12 (15.0%)	0.062	18 (15.9%)	0.486
History of alcohol use, *n* (%)	17 (15.0%)	6 (18.2%)	11 (13.8%)	0.549	20 (17.7%)	0.590
History of hypertension, *n* (%)	49 (43.4%)	14 (42.4%)	35 (43.8%)	0.897	40 (35.4%)	0.220
History of diabetes mellitus, *n* (%)	8 (7.1%)	2 (6.1%)	6 (7.5%)	0.786	11 (9.7%)	0.472

Values expressed as mean ± SD or n (%).

HAPH, high-altitude pulmonary hypertension; sPAP, systolic pulmonary artery pressure; BMI, body mass index; SBP, systolic blood pressure; DBP, diastolic blood pressure; MAP, mean arterial pressure.

*p*-values were derived from Student's t-test for normally distributed continuous variables, Mann–Whitney U test for non-normally distributed variables, and Chi–square test or Fisher's exact test (if expected values were ≤ 5) for categorical variables.

^*^P < 0.05.

The laboratory and echocardiographic characteristics of the two study populations are detailed in Table 2. Compared to non-HAPH controls, patients with HAPH exhibited significant alterations in cardiac structure and function, most evident in the enlarged dimensions of the right heart. These changes included an increase in right ventricular end-diastolic diameter, right atrial medial diameter, and right ventricular outflow tract (all *p* < 0.001). Additionally, significant differences were observed in several complete blood count parameters. The HAPH group exhibited significantly lower levels of lymphocytes, monocytes, and platelets (PLT); however, higher levels of white blood cells (WBC) compared to the non-HAPH group (all *p* < 0.05). Furthermore, the blood metabolic profile of patients with HAPH was significantly distinct, characterized by lower levels of total cholesterol (TC), triglycerides (TG), high-density lipoprotein (HDL), low-density lipoprotein (LDL), glucose (Glu), and uric acid (UA) (all *p* < 0.05). Cardiac biomarkers also differed, including N-terminal pro-B-type natriuretic peptide (NT-ProBNP) and lactate dehydrogenase, which were significantly higher in the HAPH group (both *p* < 0.001). The stratification of the HAPH population by pulmonary arterial pressure severity (sPAP ≥ 60 mmHg *vs*. 36–60 mmHg) revealed that patients with more severe HAPH (sPAP ≥ 60 mmHg) exhibited significantly larger right heart dimensions and higher NT-ProBNP levels (*p* < 0.05), indicating an association between these parameters and disease severity.

**Table 2:** The laboratory findings and echocardiographic parameters of the two study populations.

**Parameters**	**HAPH**				**Non-HAPH**	***P*-value**
	**(*****n*** = **113)**				**(*****n*** = **113)**	
	**Total**	**sPAP** ≥**60 mmHg**	**60 mmHg** > **sPAP** ≥**36 mmHg**	***P*** **-value**	**sPAP < 36 mmHg**	
	**(*****n*** = **113)**	**(*****n*** = **33)**	**(*****n*** = **80)**		**(*****n*** = **113)**	
**Cardiac function indices**						
LVEF, %	59.69 ± 10.10	57.15 ± 10.20	60.74 ± 9.94	0.086	61.81 ± 7.93	0.084
LAD, mm	35.39 ± 6.52	35.48 ± 6.38	35.35 ± 6.62	0.921	33.48 ± 5.44	0.022^*^
LVEDD1, mm	40.23 ± 7.12	40.45 ± 9.32	40.14 ± 6.05	0.453	39.65 ± 6.42	0.670
LAMD, mm	36.60 ± 7.88	34.52 ± 9.24	37.45 ± 7.14	0.052	34.82 ± 5.78	0.099
LVEDd, mm	45.24 ± 8.56	46.70 ± 11.76	44.64 ± 6.82	0.955	43.12 ± 4.84	0.328
LVPWd, mm	9.16 ± 1.72	9.42 ± 1.73	9.06 ± 1.72	0.383	8.63 ± 1.48	0.013^*^
RVEDD, mm	28.96 ± 6.50	33.45 ± 5.91	27.11 ± 5.84	< 0.001^*^	23.67 ± 2.89	< 0.001^*^
RAMD, mm	43.35 ± 8.93	48.55 ± 7.69	41.21 ± 8.55	< 0.001^*^	32.38 ± 4.37	< 0.001^*^
RVOT, mm	28.77 ± 5.09	31.58 ± 4.92	27.61 ± 4.71	< 0.001^*^	24.88 ± 2.54	< 0.001^*^
IVSd, mm	10.69 ± 1.82	10.70 ± 1.73	10.69 ± 1.86	0.983	10.23 ± 1.55	0.041^*^
**CBC indices**						
WBC, × 10^9^/L	5.70 ± 2.08	5.85 ± 1.99	5.65 ± 2.12	0.443	6.24 ± 2.02	0.017^*^
NEUT, × 10^9^/L	4.11 ± 1.96	4.28 ± 1.88	4.04 ± 2.00	0.311	4.20 ± 1.80	0.303
LYMPH, × 10^9^/L	1.11 ± 0.49	0.97 ± 0.39	1.16 ± 0.52	0.136	1.54 ± 0.64	< 0.001^*^
MONO, × 10^9^/L	0.37 ± 0.13	0.40 ± 0.15	0.36 ± 0.13	0.323	0.34 ± 0.13	0.033^*^
RBC, × 10^12^/L	4.98 ± 1.25	4.91 ± 1.04	5.01 ± 1.33	0.685	4.88 ± 1.00	0.476
HGB, g/L	136.21 ± 41.02	132.54 ± 34.67	137.74 ± 43.48	0.542	140.71 ± 35.24	0.378
PLT, × 10^9^/L	176.91 ± 78.62	180.42 ± 76.68	175.46 ± 79.84	0.752	232.01 ± 83.31	< 0.001^*^
**Blood metabolic indices**						
TC, mmol/L	3.59 ± 1.16	3.19 ± 0.83	3.76 ± 1.24	0.017^*^	4.71 ± 1.24	< 0.001^*^
TG, mmol/L	0.97 ± 0.59	0.85 ± 0.39	1.02 ± 0.65	0.241	1.55 ± 1.07	< 0.001^*^
LDL, mmol/L	2.00 ± 0.79	1.80 ± 0.59	2.08 ± 0.84	0.096	2.63 ± 0.91	< 0.001^*^
HDL, mmol/L	1.08 ± 0.75	0.92 ± 0.36	1.15 ± 0.86	0.095	1.26 ± 0.41	< 0.001^*^
Glu, mmol/L	6.05 ± 2.43	5.62 ± 1.69	6.22 ± 2.66	0.117	7.50 ± 5.19	0.031^*^
HbA1c, %	6.29 ± 1.48	6.09 ± 0.83	6.37 ± 1.67	0.531	6.92 ± 2.37	0.188
UA, μmol/L	435.80 ± 167.26	454.36 ± 158.61	428.14 ± 171.08	0.272	357.45 ± 120.81	< 0.001^*^
**Cardiac function indices**						
TnI, μg/L	0.05 ± 0.74	0.06 ± 0.09	0.04 ± 0.07	0.139	0.03 ± 0.09	0.075
LDH, U/L	514.78 ± 2,740.8	265.52 ± 123.96	617.59 ± 3,256.85	0.532	315.10 ± 1,262.98	< 0.001^*^
CK, U/L	133.24 ± 264.95	209.29 ± 460.41	101.87 ± 101.24	0.050	92.97 ± 86.55	0.266
CKMB, U/L	19.36 ± 22.86	17.23 ± 12.47	20.24 ± 25.99	0.832	14.67 ± 11.80	0.058
NT–ProBNP, pg/ml	3,519.51 ± 5,652.20	5,670.80 ± 8,004.98	2,632.11 ± 4,075.00	0.014	1,145.51 ± 8,097.46	< 0.001^*^

Values expressed as mean ± SD.

LVEF, Left ventricular ejection fraction; LAD, Left atrial diameter; LVEDD, Left ventricular end-diastolic diameter; LAMD, Left atrial medial diameter; LVEDd, Left ventricular end-diastolic dimension; LVPWd, Left ventricular posterior wall thickness at end-diastole; RVEDD, Right ventricular end-diastolic diameter; RAMD, Right atrial medial diameter; RVOT, Right ventricular outflow tract; IVSd, Interventricular septal thickness at end-diastole; CBC, Complete Blood Count; WBC, White blood cell; NEUT, Neutrophil; LYMPH, Lymphocyte; RBC, Red blood cell; HGB, Hemoglobin; PLT, Platelet; TC, Total cholesterol; TG, Total glyceride; LDL, Low density lipoprotein; HDL, High density lipoprotein; Glu, Glucose; HbA1c, Hemoglobin A1c; UA, Uric acid; TnI, Troponin I; LDH, Lactate dehydrogenase; CK, Creatine kinase; CK-MB, Creatine kinase-myocardial band; NT-ProBNP, N-terminal pro-B-type natriuretic peptide.

*p*-values were derived from Student's t-test for normally distributed continuous variables, Mann–Whitney U test for non-normally distributed variables.

^*^P < 0.05.

### 3.2 Detailed survey results of tea consumption

The detailed survey results regarding tea consumption habits are presented in Table 3. Compared to the non-HAPH group, a significantly lower proportion of patients in the HAPH group reported consuming tea (45.1% *vs*. 59.3%, *p* = 0.033). Furthermore, the HAPH population analysis demonstrated that tea consumption was even less frequent among patients with severe disease (sPAP ≥ 60 mmHg) compared to those with moderate disease (sPAP 36–60 mmHg; 27.3% *vs*. 52.5%, *p* = 0.014).

**Table 3:** Detailed survey results of tea consumption.

**Tea consumption**	**HAPH**				**Non-HAPH**	***P*-value**
	**(*****n*** = **113)**				**(*****n*** = **113)**	
	**Total**	**sPAP** ≥**60 mmHg**	**60 mmHg** > **sPAP** ≥**36 mmHg**	***P*** **-value**	**sPAP < 36 mmHg**	
	**(*****n*** = **113)**	**(*****n*** = **33)**	**(*****n*** = **80)**		**(*****n*** = **113)**	
Tea consumption, Yes/No				0.014^*^		0.033^*^
Yes	51 (45.1%)	9 (27.3%)	42 (52.5%)		67 (59.3%)	
No	62 (54.9%)	24 (72.7%)	38 (47.5%)		46 (40.7%)	
Types of tea consumption				0.022^*^		0.042^*^
No	62 (54.9%)	24 (72.7%)	38 (47.5%)		46 (40.7%)	
Green tea	14 (12.4%)	1 (3.0%)	13 (16.3%)		23 (20.4%)	
Black tea	18 (15.9%)	6 (18.2%)	12 (15.0%)		13 (11.5%)	
Tibetan tea	19 (16.8%)	2 (6.1%)	17 (21.3%)		31 (27.4%)	
Frequency of tea consumption per week				0.012^*^		0.036^*^
No	62 (54.9%)	24 (72.7%)	38 (47.5%)		46 (40.7%)	
1–3 days/week	21 (18.6%)	7 (21.2%)	14 (17.5%)		16 (14.2%)	
4–5 days/week	18 (15.9%)	1 (3.0%)	17 (21.3%)		29 (25.7%)	
≥6 days/week	12 (10.6%)	1 (3.0%)	11 (13.8%)		22 (19.5%)	
Amount of tea consumption per day				0.051		0.042^*^
No	62 (54.9%)	24 (72.7%)	38 (47.5%)		46 (40.7%)	
1–2 cups^a^/day	36 (31.9%)	7 (21.2%)	29 (36.3%)		39 (34.5%)	
≥3 cups^a^/day	15 (13.3%)	2 (6.1%)	13 (16.3%)		28(24.8%)	
Duration of tea consumption, years				0.005^*^		0.037^*^
No	62 (54.9%)	24 (72.7%)	38 (47.5%)		46 (40.7%)	
0–10 years	24 (21.2%)	8 (24.2%)	16 (20.0%)		20 (17.7%)	
10–20 years	21 (18.6%)	1 (3.0%)	20 (25.0%)		34 (30.1%)	
>20 years	6 (5.3%)	0 (0%)0	6 (7.5%)		13 (11.5%)	

Values expressed as *n* (%).

*p*-values were derived from Chi–square test or Fisher's exact test (if expected values were ≤ 5) for categorical variables.

^a^1 cup equals 200 ml.

^*^P < 0.05.

The association extended to specific patterns of tea consumption. The distribution of tea types consumed differed significantly between HAPH and non-HAPH groups (*p* = 0.042) and between the severity subgroups within the HAPH cohort (*p* = 0.022). Notably, a dose-response relationship was observed. Compared to non-HAPH controls, patients with HAPH reported a lower frequency of tea consumption per week (*p* = 0.036) and a shorter overall duration of tea drinking in years (*p* = 0.037). A similar trend of lower consumption frequency with increasing disease severity was observed within the HAPH group (*p* = 0.012). The amount consumed per serving was comparable between HAPH severity subgroups (*p* = 0.051); however, patients with HAPH overall consumed significantly less tea per serving than non-HAPH individuals (*p* = 0.042).

### 3.3 Univariate logistic regression results of baseline characteristics, laboratory findings, and echocardiographic parameters

The results of the univariate logistic regression analysis, identifying potential risk factors for PHAH, are presented in Table 4. Multiple factors exhibited significant associations. Among demographic and clinical characteristics, increasing age was a significant risk factor (OR = 1.040, 95% CI: 1.021–1.060, *p* < 0.001). In contrast, a distinct metabolic profile was strongly associated with a reduced risk, characterized by significantly lower levels of TC (OR = 0.431, 95% CI: 0.324–0.573, *p* < 0.001), LDL (OR = 0.397, 95% CI: 0.275–0.571, *p* < 0.001), TG (OR = 0.341, 95% CI: 0.207–0.561, *p* < 0.001), HDL (OR = 0.516, 95% CI: 0.276–0.963, *p* = 0.038), Glu (OR = 0.897, 95% CI: 0.821–0.979, *p* = 0.015), and glycated hemoglobin (OR = 0.837, 95% CI: 0.718–0.976, *p* = 0.023). Furthermore, PLT count (OR = 0.991, 95% CI: 0.988–0.995, *p* < 0.001) and UA levels (OR = 1.004, 95% CI: 1.002–1.006, *p* < 0.001) were significantly associated with the outcome. Factors including gender, BMI, smoking status, alcohol use, and histories of hypertension and diabetes did not exhibit significant associations in the univariate analysis.

**Table 4:** Univariate logistic regression results of baseline characteristics, laboratory findings, and echocardiographic parameters.

**Dependent variables**	**Parameter estimate**	**Standard error**	**Wald *X*^2^**	**OR (95%CI)**	***P*-value**
Age	0.040	0.010	16.507	1.040 (1.021–1.060)	< 0.001^*^
Gender	0.071	0.267	0.071	1.074 (0.636-1.814)	0.789
History of smoking	0.244	0.350	0.485	1.276 (0.643–2.534)	0.486
history of alcohol use	−0.194	0.361	0.290	0.823 (0.406–1.669)	0.590
History of hypertension	0.335	0.273	1.497	1.397 (0.818–2.388)	0.221
History of diabetes mellitus	−0.347	0.485	0.513	0.706 (0.273–1.828)	0.474
BMI	−0.035	0.028	1.507	0.966 (0.914–1.021)	0.220
MAP	0.018	0.009	3.814	1.019 (1.000–1.038)	0.051^*^
WBC	−0.130	0.068	3.666	0.878 (0.769–1.003)	0.056^*^
RBC	0.085	0.119	0.511	1.089 (0.862–1.375)	0.475
HGB	−0.003	0.004	0.780	0.997 (0.990–1.004)	0.377
PLT	−0.009	0.002	21.229	0.991 (0.988–0.995)	< 0.001^*^
TC	−0.842	0.146	33.379	0.431 (0.324–0.573)	< 0.001^*^
HDL	−0.662	0.319	4.320	0.516 (0.276–0.963)	0.038^*^
LDL	−0.925	0.186	24.677	0.397 (0.275–0.571)	< 0.001^*^
TG	−1.076	0.254	17.902	0.341 (0.207–0.561)	< 0.001^*^
Glu	−0.109	0.045	5.974	0.897 (0.821–0.979)	0.015^*^
HbA1c	−0.178	0.078	5.167	0.837 (0.718–0.976)	0.023^*^
UA	0.004	0.001	14.138	1.004 (1.002–1.006)	< 0.001^*^

^*^P < 0.2.

### 3.4 Association between tea consumption and HAPH risk

The associations between various tea consumption patterns and the risk of HAPH are detailed in Table 5. In univariate and multivariate analyses, adjusted for potential confounders (including age, MAP, WBC, PLT, lipid profiles, glucose metrics, and UA), regular tea consumption was consistently associated with a significantly lower risk of HAPH (adjusted OR = 0.496, 95% CI: 0.258–0.952, *p* = 0.035). This protective association was particularly significant for the consumption of Tibetan tea (adjusted OR = 0.300, 95% CI: 0.123–0.735, *p* = 0.008). Furthermore, a clear dose-response relationship was observed. The risk reduction was most significant in participants with the highest consumption frequency (≥6 days/week; adjusted OR = 0.208, 95% CI: 0.075–0.582, *p* = 0.003), a larger amount per day (≥3 cups; adjusted OR = 0.305, 95% CI: 0.124–0.749, *p* = 0.010), and a longer duration of consumption (≥20 years; adjusted OR = 0.210, 95% CI: 0.058–0.763, *p* = 0.018).

**Table 5:** Association between tea consumption and HAPH risk.

**Tea consumption**	**Univariate analysis**		**Multivariate analysis**	
	**OR (95%CI)**	***p*** **-value**	**Adjusted OR**^a^ **(95% CI)**	***p*** **-value**
**Regular tea consumption**				
No	Reference		Reference	
Yes	0.565 (0.333–0.957)	0.034^*^	0.496 (0.258–0.952)	0.035^*^
**Type of tea consumption**				
None	Reference		Reference	
Green tea	0.452 (0.210–0.972)	0.042^*^	0.493 (0.196–1.239)	0.133
Black tea	1.027 (0.457–2.307)	0.948	1.124 (0.411–3.073)	0.819
Tibetan tea	0.455 (0.229–0.904)	0.025^*^	0.300 (0.123–0.735)	0.008^*^
**Frequency of tea consumption per week**				
None	Reference		Reference	
1–3 days	0.974 (0.458–2.070)	0.945	0.994 (0.383–2.580)	0.991
4–5 days	0.461 (0.228–0.928)	0.030^*^	0.507 (0.219–1.178)	0.114
≥6 days	0.405 (0.182–0.901)	0.027^*^	0.208 (0.075–0.582)	0.003^*^
**Amount of tea consumption per day**				
None	Reference		Reference	
1–2 cups^b^	0.685 (0.379–1.238)	0.210	0.654 (0.313–1.367)	0.259
≥3 cups	0.397 (0.191–0.828)	0.014^*^	0.305 (0.124–0.749)	0.010^*^
**Duration of tea consumption (years)**				
None	Reference		Reference	
0–10 years	0.890 (0.440–1.803)	0.747	0.889 (0.370–2.132)	0.791
10–20 years	0.458 (0.236–0.891)	0.021^*^	0.399 (0.176–0.905)	0.028^*^
≥20 years	0.342 (0.121–0.969)	0.043^*^	0.210 (0.058–0.763)	0.018^*^

^a^ORs were adjusted for variables with a statistical significance level of ≤ 0.2 in the univariate logistic regression analysis (age, MAP, WBC, PLT, TC, HDL, LDL, TG, Glu, HbA1c, UA).

^b^1 cup equals 200 ml.

^*^P < 0.05.

## 4 Discussion

This study is the first to investigate the association between tea consumption and the risk of HAPH in high-altitude permanent inhabitants in the Tibetan population. Through a hospital-based case-control study, we observed that regular tea consumption is significantly associated with a reduced risk of HAPH. This protective effect is particularly pronounced among individuals who frequently consume Tibetan tea (≥6 days/week), drink larger quantities per day (≥3 cups), and have longer tea-drinking habits (≥20 years).

HAPH is primarily caused by a series of physiological and biochemical changes resulting from chronic exposure to hypoxia. These changes ultimately result in pathological remodeling of the pulmonary vasculature, increased pulmonary vascular resistance, and the development of pulmonary hypertension. The molecular mechanisms of HAPH have not been fully understood. Studies have reported that multiple mechanisms, including the imbalance of pulmonary vasoactive substances (including thromboxane A2, endothelin-1, prostacyclin, and nitric oxide), cellular senescence, oxidative stress, immune inflammation, and altered cellular metabolism, play important roles in the occurrence and development of HAPH (23–29). Tea has been found to possess diverse biological effects, including its ability to mitigate oxidative stress, promote anti-aging mechanisms, regulate immune function, and influence cell metabolism (17, 30, 31). The efficacy of tea in healthcare has been confirmed through multiple clinical studies involving cardiovascular, cerebrovascular, metabolic diseases, cancer, and other chronic conditions (9, 11, 12, 14, 32). However, clinical research on tea in the context of HAPH remains limited, which can be attributed to the limitations of transportation and medical resources in high altitude areas. Additionally, the relatively low prevalence of HAPH makes collecting clinical data challenging. The results of our study suggest that tea consumption can be a significant protective factor contributing to the lower incidence of HAPH in the Tibetan population. This finding is consistent with previous studies on the beneficial effects of tea on the cardiovascular system.

Furthermore, we observed a significant protective effect of Tibetan tea (adjusted OR = 0.300). However, Tibetan tea, a post-fermented dark tea, has lower catechin content and antioxidant capacity compared to green tea; it exhibits significant antioxidant activity *in vivo*. The antioxidant activity of Liubao dark tea is even more pronounced than that of certain green teas (33). In a comparative study of six types of tea, Wan et al. discovered that dark tea demonstrated superior *in vivo* activity against aging and high-fat diet-related amyloid formation activities compared to green tea. The action mechanism was associated with its antioxidant, anti-inflammatory, and lipid metabolism-improving effects (34). Under microbial fermentation, dark tea accumulates higher concentrations of organic acids, theabrownins, and flavoalkaloids. Additionally, the chemical composition of dark tea transforms with extended storage, resulting in a gradual increase in the concentration of active constituents, including tea pigments, organic acids, and tea polysaccharides. This transformation improves both the sensory quality and the bioactive efficacy of the tea (34). The protective effect of Tibetan tea against pulmonary hypertension can be attributed to its bioactive components, which confer unique benefits in high-altitude environments. Although both black tea and dark tea are classified as fully fermented teas due to their high polyphenol oxidation, they differ in fermentation mechanism: black tea undergoes endogenous enzymatic oxidation of leaf buds, while dark tea is fermented by exogenous microorganisms. This results in distinct profiles of bioactive compounds. For example, the levels of epicatechin, epigallocatechin, epicatechin gallate, and epigallocatechin gallate are significantly lower in black tea than in dark tea. Accordingly, our study found no significant association between black tea consumption and HAPH risk in either univariate or multivariate models, which may be related to the type, concentration, and biological activity of its bioactive constituents (8, 34).

A significant dose–response relationship was observed in our study: higher frequency, longer duration, and greater quantities of tea consumed per session were associated with a lower risk of HAPH. The dose–response trend is consistent with established epidemiological principles, whereby stronger and graded inverse associations may suggest a potential causal link, although causal inference in observational studies requires further investigation. It suggests that long-term and regular tea drinking habits have a cumulative effect on HAPH prevention. After adjusting for potential confounding factors, including age, blood pressure, blood routine indicators, blood lipids, Glu, and UA, the negative association between tea drinking and HAPH risk remained significant, thereby enhancing the robustness of our findings. Additionally, tea consumption in patients with HAPH exhibited a decreasing trend across disease severity subgroups, indicating that tea drinking can influence both disease incidence and severity.

For the first time, this study examines the relationship between tea consumption and HAPH in a plateau population with a distinctive cultural background and diet. All participants were Tibetan residents born in the same high-altitude area and had long-term residence, effectively controlling for variations in altitude exposure and genetic background. Additionally, we conducted a multi-dimensional assessment of tea-drinking behavior through detailed questionnaires and systematic clinical data collection.

However, this study has certain limitations. First, the case-control design cannot establish a causal relationship and is susceptible to selection bias. Second, we used TTE to evaluate sPAP. Right heart catheterization remains the gold standard for PH diagnosis, although the sPAP estimated by TTE is significantly consistent with the measured value obtained by catheterization. Third, although we controlled for a series of covariates, potential unmeasured confounding factors, including dietary structure, physical activity level, and socioeconomic status, can affect the results. In the future, it is imperative to conduct prospective cohort studies or intervention experiments to further verify the protective effect of tea consumption on HAPH. Besides, further investigation of its specific mechanisms is required, including the detection of tea polyphenol metabolites through biochemical indicators and the identification of its molecular pathways in combination with experimental studies.

In summary, this study demonstrates that regular tea consumption, particularly Tibetan tea, is independently associated with a reduced risk of HAPH and exhibits a significant dose-response relationship among the long-term high-altitude Tibetan population. These findings not only offer novel insights into the etiology of HAPH and the adaptive mechanisms of Tibetans to high-altitude environments but also provide a valuable scientific foundation for implementing diet-based primary prevention strategies in high-altitude regions. Encouraging and maintaining healthy tea consumption habits could serve as a culturally acceptable and cost-effective public health intervention, thereby contributing to the reduction of HAPH-related disease burden in this unique environmental setting.

## Data Availability

The raw data supporting the conclusions of this article will be made available by the authors, without undue reservation.
