# Supplementary material for: Tea consumption is associated with a reduced risk of high-altitude pulmonary hypertension among high-altitude permanent inhabitants in the Tibetan population: a case-control study
Source: Front Nutr. 2026 Feb 10;13:1732242. doi: 10.3389/fnut.2026.1732242 (PMC12929138; doi:10.3389/fnut.2026.1732242)
Supplement: Supplementary file 1 [file Image_1.pdf]

Supplementary Material

1.1 Supplementary Figures

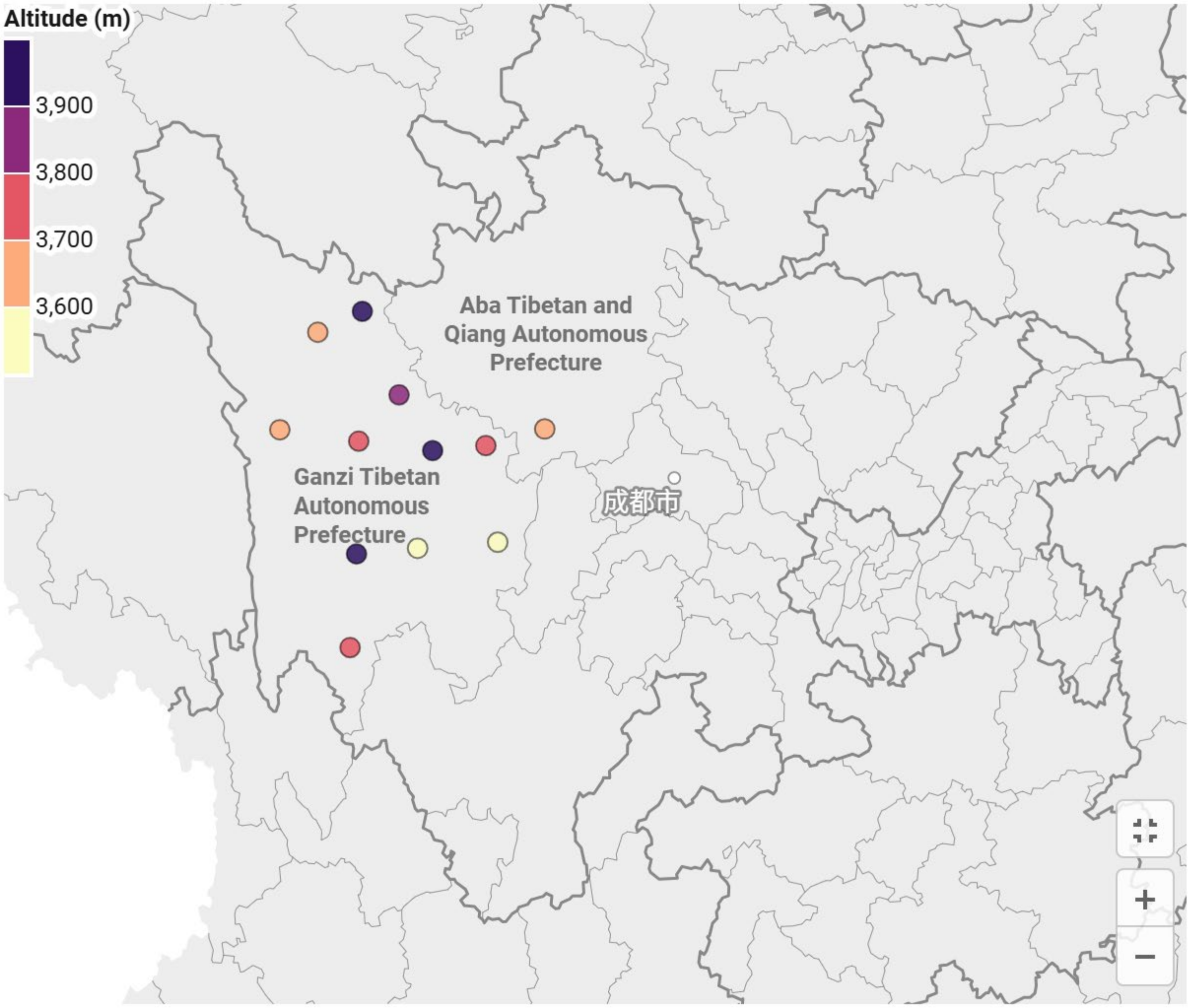

Supplementary Figure 1. Geographic distribution and altitude of participant recruitment sites.
